# Detection of rare functional variants using group ISIS

**DOI:** 10.1186/1753-6561-5-S9-S108

**Published:** 2011-11-29

**Authors:** Yue S Niu, Ning Hao, Lingling An

**Affiliations:** 1 Department of Mathematics, The University of Arizona, Tucson, AZ 85721, USA; 2 Interdisciplinary Program in Statistics, The University of Arizona, Tucson, AZ 85721, USA

## Abstract

Genome-wide association studies have been firmly established in investigations of the associations between common genetic variants and complex traits or diseases. However, a large portion of complex traits and diseases cannot be explained well by common variants. Detecting rare functional variants becomes a trend and a necessity. Because rare variants have such a small minor allele frequency (e.g., <0.05), detecting functional rare variants is challenging. Group iterative sure independence screening (ISIS), a fast group selection tool, was developed to select important genes and the single-nucleotide polymorphisms within. The performance of the group ISIS and group penalization methods is compared for detecting important genes in the Genetic Analysis Workshop 17 data. The results suggest that the group ISIS is an efficient tool to discover genes and single-nucleotide polymorphisms associated to phenotypes.

## Background

Understanding the inherited basis of genetic variation in human health and disease is currently one of the most challenging tasks. Genome-wide association studies have been used to establish the statistical association between hundreds of loci across the genome and common complex traits. Although this approach has brought substantial knowledge and understanding of the diverse molecular pathways that underlie specific diseases, more evidence shows that a large portion of complex diseases cannot be explained by common genetic variants [1,2]. Therefore alternative approaches are needed to detect and analyze rare variants associated with disease susceptibility genes. Although statistical methods for the detection of common functional variants (e.g., with minor allele frequencies [MAF] > 0.05) have been extensively developed and successively applied to numerous studies, methods for detecting rare functional variants are limited. Some methods developed for analysis of common variants can be easily extended to rare variants, for example, single-marker test, multiple-marker test, and collapsing methods, but their performance may not be optimal [3-5].

The primary purpose of this paper is to analyze quantitative traits Q1 and Q2 in replicates 1–200 of the Genetic Analysis Workshop 17 (GAW17) simulated mini-exome data [6]. We study the GAW17 data set using modern ultra-high-dimensional model selection and group selection techniques. Given the natural group structure (i.e., genes) among single-nucleotide polymorphisms (SNPs), group selection tools can select the groups that consist of a number of weak predictors (i.e., SNPs with small MAFs) whose effect as a group on the phenotypes could be significant. In the context of the GAW17 data set, these weak predictors are just rare genetic variants. Contrary to collapsing methods, modern ultra-high-dimensional model selection techniques consider the joint effect among groups as well as among individuals and avoid oversimplification of the model. We propose group iterative sure independence screening (ISIS) for gene and SNP selection. We apply the method to analyze the GAW17 data and to compare it with penalized likelihood methods, such as the group least absolute shrinkage and selection operator (LASSO) and the group minimax concave penalty (MCP) in terms of the true significant genes (i.e., genes with significant SNPs) in the simulated GAW17 data. Functional variants are referred to as important variants throughout the text.

## Methods

Because the SNPs are naturally grouped by genes, we consider a linear model with *J* groups of variables:(1)

where *y* is an *n* × 1 response vector, *X_j_* is an *n* × *p_j_* matrix corresponding to the *j*th group of variables, *β_j_* is a *p_j_* × 1 coefficient vector, and *ε* is a random noise vector with normal distribution. Denote *X* = (*X*_1_, …, *X_J_* ) and .

We assume that the model is bilevel sparse, which means that only a small number of *β_j_* are nonzero vectors and, moreover, that each nontrivial *β_j_* is itself a sparse vector. In our analysis of the GAW17 data set, the response *y* is the quantitative phenotype Q1 or Q2, and the predictors are the 24,487 SNPs grouped in 3,205 genes. The bilevel sparse assumption, interpreted in this study, says that only a small number of genes are related to the phenotype of interest and that only some of the SNPs in these related genes are important. The assumption on sparsity plays a critical role in high-dimensional statistical modeling. The bilevel sparse assumption is appropriate for models with grouped predictors.

Because the GAW17 data are mini-exome human data, we use 0, 1, and 2 to denote genotypes *AA*, *Aa*, and *aa*, respectively. Thus each column in the design matrix *X* consists of the numbers 0, 1, and 2. Among the 24,487 SNPs in the data set, there are 9,433 SNPs with a MAF of 0.07% [= 1/(697 × 2)]; that is, this is the smallest MAF in the GAW17 data because only 1 individual out of 697 individuals has a variant at each such SNP locus. The fact that 9,433 is much greater than 697 makes no statistical model identifiable. Because of the nonidentifiability of the model, it is necessary to consider the group (gene) selection.

Many high-dimensional model selection and group selection techniques have been developed recently. One of the most popular methods is the penalized likelihood method, such as the LASSO, the smoothly clipped absolute deviation (SCAD) penalty, the MCP, and their extensions for group selection (e.g., group LASSO and group MCP) [7-11]. Two popular algorithms are used to find the maximizer of the penalized likelihood. The first algorithm is the least angle regression (LARS) algorithm and its extensions [12]. It is efficient when the number of parameters (*p*) is comparable with the number of samples (*n*). However, when *p* is ultrahigh, as in our setting (24,487 potential predictors with only 697 observations), the LARS algorithm usually cannot be applied. The second algorithm is coordinate descent, which optimizes a target function with respect to a single variable at a time, iteratively cycling through all variables until convergence is reached [13]. This method is even faster than LARS and can converge to the same solutions as LARS in many cases. However, it is unknown whether the two methods work well in the ultrahigh setting.

An alternative method for model selection that is different from the penalization approach is correlation learning, for example, sure independence screening (SIS) and iterative sure independence screening (ISIS) and their extensions for the group selection, called group SIS and group ISIS [14][15]. Suppose that there are *J* groups of variables, denoted by *G*_1_, …, *G_J_*. In the first step of this approach, regression is performed using a single group of variables for each *G_j_*, *j* = 1, …, *J*. (Here, if the number of variables in *G_j_* is small, we can simply perform a least-squares regression. Otherwise, when the size of *G_j_* is large, we can use penalized regressions [LASSO or SCAD] or greedy algorithms [forward stepwise selection] to select the model and to estimate the coefficients.) After the first step, the residual sum of squares (RSS) is calculated by dividing by the degrees of freedom (df) for each regression using a single group. Then, the groups are ranked, and the top *k* groups are selected on the basis of the smallest RSS/df value. This is the main procedure for the group SIS method.

The group ISIS is just the iterative version of group SIS. First, one or several groups, say, *G_i_*_1_, …, *G_ik_*, are selected using group SIS. Then, regression is performed using these selected groups together with a single new group from the rest of the groups for each of the new groups. Next, all the new groups are ordered by the RSS/df values, and top groups with the smallest RSS/df values are selected and added to the pool of selected groups. This process is iterated until some criterion is met. Note that this version of group ISIS corresponds to a version of ISIS introduced by Fan et al. [15], which improves the original ISIS given by Fan and Lv [14].

Group SIS and group ISIS are general strategies, and they have many variants in different applications. In this paper, we describe two procedures related to group ISIS for gene selection and apply them to analyze the GAW17 data. In the GAW17 data, the phenotypes may have strong associations with factors such as sex, age, and smoking. Therefore these factors are always considered important by default.

For procedure 1 we preset a maximal iteration *K* (12 or 15 in our study) and a constant *C* (5 in our simulation). In the first step, we perform a regression using each single group *G_j_* and sex, age, and smoking, where *j* goes from 1 to *J*. We restrict the model size to at most *C* + 3 when we do the regressions. For example, if the *j*th group *G_j_* consists of 12 variables, we use a forward stepwise selection method to select 5 among those 12 variables besides sex, age, and smoking. Then, we rank the RSS/df values for all groups. After ranking, we select the group, denoted by *G*_(1)_, with the lowest RSS/df value in the model. At the *k*th iteration, we perform a regression using selected groups *G*_(1)_, …, *G*_(_*_k_*_−1)_, sex, age, and smoking together with a single group for each group from the rest. We restrict the whole model size to at most *Ck* + 3 when we do the regressions. Then, we select the new group *G*_(_*_k_*_)_ by ranking the RSS/df values. The procedure ends when *k* >*K*. In short, we select one group each time until the *k*th iteration. As we will see, this procedure can be considered a special case of procedure 2. However, as the counterpart of forward stepwise selection in the setting of group selection, procedure 1 is an interesting procedure in itself. Therefore we include it in our numeric studies.

Procedure 2 is similar to procedure 1 except that it selects multiple groups each time and allows deletion. To make the algorithm more efficient, we select *k*_0_ = 5 groups with the lowest RSS/df values at each iteration. After adding these five new groups, we do group selection immediately and keep only the selected groups before entering the next iteration. The procedure stops when it is stable, that is, when it deletes newly selected groups in the group selection. We use group LASSO and group MCP with either the Akaike information criterion (AIC) or the Bayesian information criterion (BIC) for group selection in the deletion step and discard the groups that are not selected by group LASSO or group MCP. We find that the procedure usually stops within 10 iterations, so we set the maximum number of iterations at 10. Group MCP and group LASSO are implemented using R package grpreg and can also be directly applied to select important genes. It is interesting to compare procedure 1 and procedure 2 with these methods.

## Results

Two phenotypes of Q1 and Q2 are studied as interesting responses in this paper. Because there are 200 replicates of data sets in GAW17 and because each replicate has a certain amount of noise, we consider three noise levels (low, medium, and high). In the low noise level case, we take average of 200 replicates as the response variable; the medium noise level refers to the average of 10 replicates. For the high noise level, only one replicate is considered. We select the first replicate as an example of high noise data to compare the different selection methods. Other individual replicates could have been used, but the same conclusion is obtained.

Both group ISIS procedures (procedure 1 and procedure 2) are used for gene selection. In particular, for procedure 2 we apply group MCP and group LASSO by means of the AIC and BIC in the deletion step of each iteration. We also compare procedure 1 and procedure 2 with the penalization approaches (group MCP and group LASSO). The results are listed in Table 1. One can see that group ISIS can select most of the important genes with a small number of false positives from a huge pool when the noise level is low or medium.

**Table 1:** Comparisons of penalized likelihood methods and group ISIS procedures 1 and 2 at three different noise levels

Noise level	Penalized likelihood method				Group ISIS procedure 1	Group ISIS procedure 2			
			
	Group MCP (with AIC)	Group MCP (with BIC)	Group LASSO (with AIC)	Group LASSO (with BIC)		Group MCP (with AIC)	Group MCP (with BIC)	Group LASSO (with AIC)	Group LASSO (with BIC)
Q1									
Low	45/4	8/4	168/1	168/1	12/5	8/5	5/4	4/4	4/4
Medium	89/5	10/4	216/1	147/1	12/5	9/5	7/5	5/5	3/3
High	233/3	121/3	233/1	177/1	12/2	24/2	22/2	13/2	13/2
Q2									
Low	41/12	9/6	101/6	37/6	15/12	13/12	7/7	11/11	9/7
Medium	55/11	11/6	176/4	74/3	12/9	19/8	8/6	20/8	12/10
High	250/8	125/5	259/1	259/1	15/0	35/1	33/1	19/1	24/1

In group ISIS procedure 2, penalized likelihood methods (group LASSO and group MCP) were used in the deletion step. The number of selected genes and the number of selected important genes (true positives) are reported.

To compare these methods more carefully, we applied them to 20 data sets with medium noise level. The data were obtained by taking the average of replicates 1–10, 11–20, …, 191–200. The results are summarized in Table 2. It seems that the performance of the penalized likelihood methods highly depends on the penalty structure (group LASSO or group MCP) and information criterion (AIC or BIC). Group MCP with BIC performed best among all four methods we investigated. We see that group ISIS procedure 2 performed well with lower numbers of false positives and lower false discovery proportions compared with penalized likelihood methods. Furthermore, based on the result of group ISIS, penalized likelihood methods or other methods can be applied in the second stage to select important SNPs. In fact, as a by-product in the final iteration of procedure 2, group MCP does perform SNP selection within selected genes (see Table 3). For example, in the case of low noise level Q2, The group MCP procedure 2 with AIC selected 13 genes with one false positive (*OR1Q1*) and one false negative (*INSIG1*). The procedure became stable at iteration 5. In the final deletion step, group MCP with AIC selected 13 genes among 18 and 32 SNPs within these 13 selected genes. We found only 3 false-positive SNPs (2 of them from *OR1Q1* and 1 from *SREBF1*) among these 32 SNPs. Group MCP is quite aggressive for the individual selection within groups. One could use other methods to lower the number of false negatives regarding SNP selection.

**Table 2:** Comparisons of penalized likelihood methods and group ISIS procedure 2 at medium noise level over 20 data sets

Noise level	Penalized likelihood method				Group ISIS procedure 2			
	
	Group MCP (with AIC)	Group MCP (with BIC)	Group LASSO (with AIC)	Group LASSO (with BIC)	Group MCP (with AIC)	Group MCP (with BIC)	Group LASSO (with AIC)	Group LASSO (with BIC)
Q1								
NTP	4.2 (0.4)	3.7 (0.6)	1.1 (0.4)	1.1 (0.3)	4.3 (0.5)	3.8 (0.5)	4.3 (0.6)	3.0 (0.9)
NFP	86.1 (6.2)	7.0 (1.9)	205.6 (23.8)	117.2 (20.4)	5.0 (1.8)	1.1 (0.9)	2.1 (2.8)	0.1 (0.3)
FDP (%)	95.3 (0.6)	64.2 (9)	99.5 (0.2)	99.0 (0.3)	52.2 (10.7)	18.8 (14.1)	21.9 (26.5)	2.1 (8.3)
Q2								
NTP	10.9 (0.9)	6.3 (0.6)	3.5 (1.3)	3.2 (1.3)	8.4 (1.2)	5.8 (1.5)	8.6 (1.6)	8.2 (1.8)
NFP	55.7 (6.8)	7.5 (2.2)	160.3 (30.0)	99.0 (27.1)	10.8 (3.7)	2.3 (1.3)	10.6 (2.0)	4.2 (2.5)
FDP (%)	83.5 (2.1)	53.2 (9.2)	97.7 (1.1)	96.8 (1.4)	55.0 (9.8)	27.9 (14.8)	55.1 (6.3)	31.6 (15.0)

Reported are the mean and standard error (in parentheses) of the number of true positives (NTP), the number of false positives (NFP), and the false discovery proportion (FDP).

**Table 3:** Genes and SNPs selected by group ISIS procedure 2 (group MCP with AIC) for the low noise data

Trait	Selected gene	Selected SNPs
Q1	*FLT1*	C13S348*, C13S431, C13S522, C13S523, C13S524
	LRP1B*	C2S3362*
	*KDR*	C4S1861, C4S1878, C4S1884
	PRR4*	C12S706*
	*ARNT*	C1S6533, C1S6542
	*VEGFA*	C6S2981
	*HIF1A*	C14S1734
	C20ORF26*	C20S640*
Q2		
	VNN3	C6S5441, C6S5446, C6S5449
	*VNN1*	C6S5378, C6S5380
	*BCHE*	C3S4859, C3S4869, C3S4873, C3S4874, C3S4875
	*SIRT1*	C10S3048, C10S3050
	*SREBF1*	C17S1019*, C17S1024, C17S1043, C17S1046, C17S1055
	*VLDLR*	C9S376, C9S377, C9S444
	*PDGFD*	C11S5292, C11S5301, C11S5302
	*GCKR*	C2S354
	*LPL*	C8S442, C8S530
	*PLAT*	C8S1741, C8S1758
	*VWF*	C12S211
	*OR1Q1*	C9S3735*, C9S3737*
	*RARB*	C3S679

Asterisks represent false positives.

Although it is unlikely in practice, when additional information is known in advance (e.g., all important SNPs are nonsynonymous or have MAF less than 0.2), we can filter out parts of the SNPs first and apply group ISIS to the rest. There are 13,572 nonsynonymous SNPs in 2,196 genes and 23,131 low-MAF (<0.2) SNPs in 3,100 genes. For the phenotypes Q1 and Q2, all important SNPs are nonsynonymous and have low MAF, so we lose nothing by searching among the restricted data set. We applied group ISIS to these data sets and found that the results were almost identical with the results reported in Table 1. Therefore we conclude that ultra-high dimensionality and existence of common variants (say, MAF > 0*.*2) hardly influence the performance of group ISIS. That is one of the main advantages of group ISIS.

The high noise level is the main reason that no method works well using only one replicate of the data. Besides that, to understand why some genes (e.g., *VEGFC* for Q1) cannot be selected even for the low noise data, we checked the data carefully. We found that SNP C4S4935, the only important SNP in gene *VEGFC*, is identical to SNP C13S348 in gene *FLT1* in the design matrix. Gene *FLT1* includes many SNPs related to Q1. So if *FLT1* is selected, *VEGFC* does not have any priority to be selected. In short, the nonidentifiability may bring up some issue in gene selection when some important genes have few related SNPs.

## Discussion and conclusions

In this study, we used modern ultra-high-dimensional model selection tools to detect important genes and important SNPs related to the phenotypes of interest. Group SIS and group ISIS were developed to conquer the difficulty of ultra-high dimensionality and nonidentifiability. As group LASSO is to LASSO, group SIS (or group ISIS) is the counterpart to SIS (ISIS) in the setting of group selection. These group selection tools work well under the bilevel sparse assumption. We used the penalized likelihood methods and group ISIS procedures to analyze the GAW17 data and compared their performance in recovering important genes associated with phenotypes Q1 and Q2. It seems that the group ISIS approach performs better than the penalized likelihood methods in terms of number of false positives and false discovery proportion. In particular, the proposed methods work well when additional replicates are available, that is, at the low and medium noise levels. When only one replicate with high noise is available, it seems that no method works well.

## Competing interests

The authors have no conflict of interest.

## Authors’ contributions

YSN designed the study, performed the statistical analysis, and drafted the manuscript. NH participated in its design and statistical analysis, and helped to refine the statistical procedures. LA conceived of the study, and participated in its design and coordination and helped to draft the manuscript. All authors read and approved the final manuscript.

## References

[B1] McCarthy MI Abecasis GR Cardon LR Goldstein DB Little J Ioannidis JPA Hirschhorn JN Genome-wide association studies for complex traits: consensus, uncertainty, and challenges Nat Rev Genet 2008 9 356 369 10.1038/nrg2344 18398418

[B2] Nejentsev S Walker N Riches D Egholm M Todd JA Rare variants of *IFIH1*, a gene implicated in antiviral responses, protect against type 1 diabetes Science 2009 324 387 389 10.1126/science.1167728 19264985 PMC2707798

[B3] Li B Leal SM Methods for detecting associations with rare variants for common diseases: application to analysis of sequence data Am J Hum Genet 2008 83 311 321 10.1016/j.ajhg.2008.06.024 18691683 PMC2842185

[B4] Matthews AG Haynes C Liu C Ott J Collapsing SNP genotypes in case-control genome-wide association studies increases the type I error rate and power Stat Appl Genet Mol Biol 2008 7 article 23 10.2202/1544-6115.1325 PMC2789285 18673292

[B5] Xiong M Zhao J Boerwinkle E Generalized T2 test for genome association studies Am J Hum Genet 2002 70 1257 1268 10.1086/340392 11923914 PMC447600

[B6] Almasy LA Dyer TD Peralta JM Kent JW Jr. Charlesworth JC Curran JE Blangero J Genetic Analysis Workshop 17 mini-exome simulation BMC Proc 2011 5 suppl 9 S2 22373155 10.1186/1753-6561-5-S9-S2 PMC3287854

[B7] Breheny P Huang J Penalized methods for bi-level variable selection Stat Interface 2009 2 369 380 20640242 10.4310/sii.2009.v2.n3.a10 PMC2904563

[B8] Fan J Li R Variable selection via nonconcave penalized likelihood and its oracle properties J Am Stat Assoc 2001 96 1348 1360 10.1198/016214501753382273

[B9] Tibshirani R Regression shrinkage and selection via the LASSO J R Stat Soc Ser B 1996 58 267 288

[B10] Yuan M Lin Y Model selection and estimation in regression with grouped variables J R Stat Soc Ser B 2006 68 49 67 10.1111/j.1467-9868.2005.00532.x

[B11] Zhang CH Nearly unbiased variable selection under minimax concave penalty Ann Stat 2010 38 894 942 10.1214/09-AOS729

[B12] Efron B Hastie T Johnstone I Tibshirani R Least angle regression Ann Stat 2004 32 407 499 10.1214/009053604000000067

[B13] Friedman J Hastie T Hoffling H Tibshirani R Pathwise coordinate optimization Ann Appl Stat 2007 1 302 332 10.1214/07-AOAS131

[B14] Fan J Lv J Sure independence screening for ultrahigh dimensional feature space J R Stat Soc Ser B 2008 70 849 911 10.1111/j.1467-9868.2008.00674.x PMC2709408 19603084

[B15] Fan J Samworth R Wu Y Ultrahigh dimensional feature selection: beyond the linear model J Mach Learn Res 2009 10 2013 2038 21603590 PMC3095976

